# Genomic insights into *Brucella melitensis* in India: stability of ST8 and the role of virulence genes in regional adaptations

**DOI:** 10.1128/spectrum.02647-24

**Published:** 2025-04-24

**Authors:** Haris Ayoub, M. Suman Kumar, Rishabh Mehta, Sanjumon E. Sethuraj, Prasad Thomas, Himani Dhanze, Muskan Dubey, Harith M. Salih, Girish B. Chandrashekaraiah, Charley A. Cull, Ravindra P. Veeranna, Raghavendra G. Amachawadi

**Affiliations:** 1 Division of Veterinary Public Health, ICAR-Indian Veterinary Research Institute 30072, Izatnagar, Uttar Pradesh, India; 2 Division of Bacteriology and Mycology, ICAR-Indian Veterinary Research Institute 30072, Izatnagar, Uttar Pradesh, India; 3 Xavier University School of Medicine, Xavier University School of Veterinary Medicine, Oranjestad, Aruba; 4 Department of Clinical Sciences, College of Veterinary Medicine, Kansas State University 5308, Manhattan, Kansas, USA; 5 Veterinary College, Hassan, Karnataka, India; 6 Midwest Veterinary Services, Inc, Oakland, New Jersey, USA; UJF-Grenoble 1, CHU Grenoble, Grenoble, France

**Keywords:** *Brucellosis*, phylogeny, pangenome, MSLT

## Abstract

*Brucella melitensis* is a highly infectious zoonotic pathogen responsible for brucellosis, which significantly affects both human and livestock health worldwide. This study employed whole-genome sequencing (WGS) to analyze the genetic diversity of 24 *B. melitensis* isolates from India. Pangenome analysis revealed a highly conserved nature with the involved strains having very limited accessory genes. Multilocus Sequence Typing (MLST) identified sequence type ST8 as predominant among Indian strains. Analysis of virulence genes revealed a total of 43 virulence-related genes in all strains, emphasizing their critical role in the pathogenicity of *B. melitensis*. Unique gene profiles and distinct phylogenetic clusters suggest regional adaptations and evolutionary pressures. The comprehensive genomic insights from this study help to elucidate the geographic distribution and interspecies transmission of Indian strains, highlighting the importance of targeted brucellosis control measures in India. Additionally, the identification of conserved virulence genes involved in immune evasion and intracellular survival highlights their importance in the bacterium’s pathogenicity. This research contributes to the global understanding of *B. melitensis* genomic diversity, providing valuable insights for broader epidemiological studies and brucellosis management strategies worldwide.

**IMPORTANCE:**

*B. melitensis* is a significant cause of illness in both humans and animals, particularly in India, where the disease remains a major concern. This study highlights that only a few genetic types of the bacteria are circulating in the region, which means control efforts can be better focused on these specific types. By understanding the unique characteristics of Indian strains, and how these strains spread and adapt, this research offers valuable guidance for improving brucellosis prevention strategies. These insights can help in developing more effective diagnostic tools, enhancing vaccination efforts, and strengthening disease control programs to reduce the impact of brucellosis on public health and livestock industries.

## INTRODUCTION

*Brucella melitensis* is a highly infectious zoonotic pathogen responsible for brucellosis, a disease that significantly affects both humans and livestock. Brucellosis remains a significant public health concern globally, with an estimated 2.1 million new human infections annually (1). *B. melitensis*, the most virulent species for humans, primarily affects goats and sheep (2), with zoonotic transmission occurring through the ingestion of unpasteurized dairy products or direct contact with infected animal tissues (3). Despite its significant impact, brucellosis is often underreported and misdiagnosed, particularly in endemic regions, due to its nonspecific clinical presentation, which includes fever, malaise, and musculoskeletal pain (4). Brucellosis is particularly prevalent in developing countries, including India, where it poses substantial public health and economic challenges (5). The pathogen is primarily transmitted through direct contact with infected animals or the consumption of contaminated animal products, such as unpasteurized milk (3). The epidemiology of *B. melitensis* in India is complex, involving multiple transmission routes and diverse animal reservoirs (4).

Understanding the genetic diversity and phylogenetic relationships of *B. melitensis* is crucial for effective disease control and prevention. Phylogenetic studies provide insights into the evolutionary history and geographical distribution of the pathogen, aiding in the identification of outbreak sources and transmission pathways (6). Recent advancements in whole-genome sequencing (WGS) have significantly enhanced our understanding of the genetic diversity and evolution of *B. melitensis*. WGS allows for comprehensive analysis of the entire genome, identifying single-nucleotide polymorphisms (SNPs), insertions, deletions, and other genetic variations (3).

Traditional methods of studying genetic diversity, such as phage typing and biotyping based on biochemical and serological characteristics, have limitations in their discriminatory power and reproducibility (7). In contrast, molecular techniques like MLST offer higher resolution and have become the gold standard for epidemiological studies (8). MLST characterizes strains based on the sequences of multiple housekeeping genes, allowing for the assignment of sequence types (STs) and facilitating comparison across different studies and geographical regions (9). Pangenome analysis extends this approach by comparing the entire genome content of multiple strains, identifying core and accessory genes, and elucidating the genetic basis of phenotypic diversity. This approach provides insights into the genetic repertoire of the species and the mechanisms underlying its adaptability and pathogenicity (10–12).

Whole-genome phylogeny uses WGS data to construct phylogenetic trees that reveal the evolutionary relationships among strains. This method provides a high-resolution view of genetic relationships and can identify clades and lineages with precision (13, 14). Combining WGS with MLST, pangenome analysis and whole-genome phylogeny enable a detailed understanding of the genetic structure and epidemiology of *B. melitensis* (14–16).

In India, brucellosis is endemic in many regions, with significant implications for public health and livestock productivity. Despite this, there is a paucity of data on the genetic diversity of *B. melitensis* strains circulating in the country. Previous studies have primarily focused on seroprevalence and clinical aspects, with limited molecular epidemiological investigations (4, 11, 17–22). Understanding the genetic diversity of *B. melitensis* in India is crucial for developing effective control strategies and preventing the spread of the disease (21). By identifying the genetic diversity and transmission dynamics of *B. melitensis*, we can develop more targeted diagnostic tools and vaccines, improve surveillance and control measures, and ultimately reduce the burden of brucellosis in India.

This study aims to analyze the genetic diversity of *B. melitensis* strains isolated from humans and livestock in India using a combination of MLST, pangenome analysis, and whole-genome phylogeny. By employing these advanced molecular techniques, we seek to elucidate the genetic relationships among these strains and provide a comprehensive overview of the epidemiological landscape of *B. melitensis* in India.

## MATERIALS AND METHODS

### Selection of isolates

*B. melitensis* isolates used in this study were obtained from a previously established collection of strains submitted for confirmation and biotyping at the Brucella Laboratory, Division of Veterinary Public Health, ICAR-IVRI, Izatnagar, Uttar Pradesh. This study analyzed a total of 24 isolates collected from various regions across India between 2006 and 2023. These isolates comprised 20 from humans, 1 from a goat, and 3 from sheep. Initial cultivation of these isolates involved inoculating samples onto Brucella agar and incubating them at 37°C under 10% CO_2_ for up to 7 days. The isolates underwent identification through Gram staining, biochemical tests, and dye inhibition tests, following the established protocols (23). Species confirmation was achieved using AMOS PCR (24), with *B. melitensis* 16M (ATCC 23456; NCTC 10094) serving as the reference positive control strain.

### Extraction of genomic DNA from isolates

For genome sequencing, genomic DNA was extracted from 23 recent subcultures of *B. melitensis* isolates using the QIAamp DNA Mini Kit (QIAGEN), following the manufacturer’s protocol with nuclease-free water (NFW) as the solvent. The concentration and purity of the extracted DNA were assessed by measuring its optical density (OD) at 260 and 280 nm using a spectrophotometer (Eppendorf BioSpectrometer Basic- EP6135000923). The purified DNA samples were subsequently shipped on dry ice for whole-genome sequencing.

### Genome sequencing, assembly, and annotation

Genome sequencing of the 23 isolates (excluding VPH-17-72) was carried out on the Illumina MiSeq platform, generating 150 bp paired-end reads (miBiome Therapeutics LLP). Whole-genome sequence of the isolate VPH-17-72 (Goat origin) was retrieved from the NCBI database and used in the study. The quality of the raw reads was assessed using FastP v0.23.2 (25). The high-quality trimmed reads were then assembled *de novo* using Unicycler v0.5.0 (26) to construct genome representing contigs.

The quality of the genome assemblies was evaluated using QUAST v5.2.0 (27), which analyzed metrics such as N50 (the contig length at which half of the genome is covered), contig size, and the number of uncalled bases (Ns). The completeness of the genome assemblies was further assessed using the Benchmarking Universal Single-Copy Orthologs (BUSCO v5.4.6) tool (28). For gene prediction, functional annotation, and feature identification, the NCBI Prokaryotic Genome Annotation Pipeline (29) was employed. Most of the bioinformatics analysis was conducted on the Galaxy Europe platform (usegalaxy.org) (30). The genome sequence of *B. melitensis* bv 1 str. 16M served as the reference for ordering the contigs and ensuring the accuracy of the assembled genomes.

### Comparative genomic analysis

#### Data retrieval

The comparative genomic analysis encompassed the 24 study isolates (including VPH-17-72) of *B. melitensis* from humans and livestock in India, collected between 2006 and 2023. Genome data for 38 *B. melitensis* strains were downloaded from the NCBI database, encompassing various regions, including India, China, Malaysia, Saudi Arabia, and Kuwait. Details such as host, geographical location, year of isolation, and accession numbers were noted for all strains to provide comprehensive contextual information for comparative analysis (Table 1).

**TABLE 1:** Metadata of 38 *B. melitensis* isolates retrieved from NCBI for phylogeny

S. no	Strain	Assembly accession	Place	Source	Date of collection	Host
1	M5-10	GCA_000292065.1	China	Vaccine strain	NA^*a*^	NA^*a*^
2	BCB033	GCA_000292085.2	China	Blood	2006	Human
3	BCB028	GCA_000292165.2	China	Blood	1956	Sheep
4	133	GCA_000298595.1	China	Blood	1998	Human
5	128	GCA_000298615.1	China	Serum	1986	Human
6	ADMAS-G1	GCA_000444515.1	India	Placenta	2013	Goat
7	BRUC048	GCA_001608355.1	Egypt	Blood	NA^*a*^	NA^*a*^
8	KU_RCF-96	GCA_001702255.1	Kuwait	Blood	2014	Human
9	KU_RCF-84	GCA_001702305.1	Kuwait	Blood	2014	Human
10	KU_RCF-03	GCA_001702375.1	Kuwait	Blood	2014	Human
11	Br-m-1771_12-Geo	GCA_001856285.1	Georgia	Blood	2012	Human
12	Br-m-1252_10-Geo	GCA_001856295.1	Georgia	Milk	2010	Cattle
13	Br-m-1268_11-Geo	GCA_001856365.1	Georgia	Milk	2011	Cattle
14	VRI-6856_11	GCA_002245205.1	Malaysia	Milk	2011	Goat
15	VRI4799_15	GCA_002245235.1	Malaysia	Milk	2015	Goat
16	2007BM_1	GCA_002290125.1	India	Blood	2007	Human
17	CIIMS-NV-5	GCA_002871075.1	India	Vaginal discharge	2016	Goat
18	CIIMS-BH-2	GCA_002895105.1	India	Blood	2016	Human
19	CIIMS-PH-3	GCA_002895125.1	India	Blood	2016	Human
20	Rev-1_passage101	GCA_002953595.1	USA	NA^*a*^	1970	Small ruminants
21	CIIMS-NV-1	GCA_003205535.1	India	NA^*a*^	2016	Animal
22	KSA_BM_07	GCA_003432005.1	Saudi Arabia	Fetal fluids	2017	Sheep
23	CIT21	GCA_003516045.1	China	Cell culture	2015	Human
24	CIT31	GCA_003516065.1	China	Cell culture	2015	Human
25	CIT43	GCA_003516085.1	China	Cell culture	2015	Human
26	LMN19	GCA_003989635.1	India	Placenta	2018	Goat
27	LMN20	GCA_003989875.1	India	Placenta	2018	Goat
28	LMN18	GCA_003989885.1	India	Foetal stomach content	2018	Goat
29	LMN17	GCA_003989895.1	India	Foetal stomach content	2018	Goat
30	2011-TE-13541-1-1	GCA_006517325.1	Italy	NA^*a*^	2011	Goat
31	2016-TE-17270-1-1	GCA_006517335.1	Italy	NA^*a*^	2016	Sheep
32	TN_CUL_1	GCA_014270005.1	India	Fetal stomach content	2017	Sheep
33	QH2019001	GCA_016411965.1	China	Blood	2019	Human
34	QH2019005	GCA_016806105.1	China	Blood	2019	Human
35	BMNDDB8664	GCA_022348405.1	India	Vaginal swab	2019	Cattle
36	BRC9_11	GCA_028129055.1	Malaysia	Blood	2011	Human
37	BRC5_11	GCA_028129055.1	Malaysia	Blood	2011	Human
38	BRC27_11	GCA_028129085.1	Malaysia	Blood	2011	Human

^*a*^ Not available.

#### Pangenome analysis

Pangenome analysis was conducted using Panaroo (31) to explore the genetic diversity and identify core and accessory genes within the *B. melitensis* strains. The results were visualized to illustrate the distribution of gene presence and absence among the isolates. This analysis provided insights into the genetic repertoire of the species, including genes that contribute to its adaptability and pathogenicity. Two-dimensional scaling and visualization of the pangenome were performed using FriPan (https://github.com/drpowell/FriPan), facilitating the examination of genetic differences and similarities among the isolates.

#### MLST & virulence genes

Multilocus Sequence Typing (MLST) was employed to genotype the *B. melitensis* isolates. Genotyping was performed using the PubMLST*—Brucella* database (https://pubmlst.org/), applying both nine-locus (9) and 21-locus (32) schemes, as well as the cgMLST scheme (33) to characterize the isolates based on their sequence types, facilitating comparisons across different studies. Virulence factors were predicted using ABRicate searches of assembled contigs against the Virulence Factor Database (VFDB) with a minimum 90% DNA identity and coverage (https://github.com/tseemann/abricate) (34, 35). This analysis identified genes associated with pathogenicity and virulence.

#### Phylogeny and global clustering

To infer the phylogenetic relationships among the *B. melitensis* strains, whole-genome alignment was performed using parSNP v1.2 (https://github.com/marbl/parsnp) (36). The output from parSNP was converted into a multiple sequence alignment in FASTA format, which was then transformed into phylip format. The resulting alignment was used to construct a phylogenetic tree.

For global clustering, the optimal model and method were identified, and a phylogenetic tree was constructed using RAxML-NG v1.2.0, employing the maximum likelihood approach with the GTR + G substitution model. An initial tree search with 10 parsimony trees was conducted, followed by 200 bootstrap iterations to evaluate the robustness of the inferred phylogeny (37). The phylogenetic tree was visualized and annotated with relevant metadata using iTOL (38). This analysis provided insights into the global phylogenetic relationships and clustering of Indian *B. melitensis* strains among global strains, revealing their evolutionary history and spread patterns.

#### Minimum spanning tree analysis

Core genome multilocus sequence typing (cgMLST) was performed using PyMLST (39), an open-source tool for rapid MLST assignment. A total of 63 *Brucella melitensis* strains were included in the analysis, comprising 36 Indian isolates (24 study isolates and 12 from NCBI) and 27 global isolates. The cgMLST scheme for *B. melitensis* was applied, identifying 1,763 core genes from a total of 1,764 loci across all strains. To assess genetic relatedness, a Minimum Spanning Tree (MST) was constructed using GrapeTree (MSTreeV2) (40). The MST was generated based on allele differences between strains. A minimum coverage threshold of 50 strains (-m 50) was applied to retain genes found in at least 50 isolates, ensuring robust cluster formation and minimizing missing data artifacts.

## RESULTS

### Virulence genes

The Virulence Factor Database (VFDB) identified a total of 43 virulence-related genes across all tested strains of *Brucella melitensis* (Table 2). The majority of these genes were part of the lipopolysaccharide (LPS) operon, comprising 27 genes that play crucial roles in entry, intracellular survival, and immunomodulation. Additionally, 12 genes associated with the type IV secretion system (*virB1-virB12*) were identified, which are vital for effector secretion. Other significant virulence genes included *btpA* and *btpB* (TIR domain-containing proteins involved in immune evasion), *ricA* (Rab2 interacting conserved protein A for intracellular survival), and *cgs* (cyclic beta 1–2 glucan synthetase for intracellular survival).

**TABLE 2:** Virulence and pathogenicity factors identified in *B. melitensis* strains

Virulence and pathogenicity factors	Function	Genes
LPS (lipopolysaccharide) pathogenicity factors	Entry, intracellular, survival, and immunomodulatory	*acpXL, fabZ, gmd, htrB, kdsA, kdsB, lpsA, lpsB/lpcC, lpxA, lpxB, lpxC, lpxD, lpxE, manAoAg, manCoAg, per, pgm, pmm, wbdA, wbkA, wbkB, wbkC, wboA, wbpL, wbpZ, wzm, wzt*
Type IV secretion system	Effector secretion	*virB1, virB2, virB3, virB4, virB5, virB6, virB7, virB8, virB9, virB10, virB11, virB12*
TIR domain-containing protein	Immune evasion	*btpA, btpB*
Rab2 interacting conserved protein A	Intracellular survival	*RicA*
Cyclic beta 1–2 glucan synthetase	Intracellular survival	*Cgs*

### Multilocus sequence typing

Multilocus sequence typing (MLST) based on the 9-locus and 21-locus schemes identified all *Brucella* isolates as sequence type ST8. The core genome MLST (cgMLST) scheme revealed five different sequence types among the study isolates, demonstrating some genetic diversity within this species (Table 3).

**TABLE 3:** Multilocus sequence analysis and cgMLST results for *B. Melitensis* isolates

Strain	MLST9 scheme	MLST21 scheme	cgMLST	Loci matched
16M	7	7	218	
VPH-06-01	8	8	557	99.40%
VPH-08-01	8	8	557	100%
VPH-08-02	8	8	225	97.40%
VPH-17-72	8	8	573	100%
VPH-19-01	8	8	670	98.50%
VPH-19-02	8	8	670	99.20%
VPH-19-03	8	8	670	95%
VPH-19-04	8	8	225	96.40%
VPH-19-05	8	8	670	98.30%
VPH-19-06	8	8	557/565	98.40%
VPH-19-07	8	8	670	99.20%
VPH-20-01	8	8	670	98.10%
VPH-20-02	8	8	670	99.70%
VPH-20-03	8	8	670	98.30%
VPH-20-04	8	8	670	98.40%
VPH-21-01	8	8	557/565	98.80%
VPH-21-02	8	8	557	98.70%
VPH-22-01	8	8	557	98.30%
VPH-22-02	8	8	557	98.6%
VPH-22-03	8	8	670	97.80%
VPH-22-04	8	8	670	99.50%
VPH-22-05	8	8	565	98.90%
VPH-23-01	8	8	670	99.30%
VPH-23-02	8	8	670	99.30%

### Pangenome and phylogeny

The pangenome analysis of the *B. melitensis* isolates revealed a highly conserved core genome structure, consisting of 2,899 core genes and an additional 185 soft core genes among a total of 3,150 genes (Table 4). This indicates a significant degree of genetic conservation within the species. One isolate, VPH-19-03, displayed about 6% missing genes and a genome fraction of only 94% compared to the reference, suggesting some unique genetic variations.

**TABLE 4:** Pangenome summary of study isolates

Genes	Gene coverage	No. of genes identified
Core genes	(99% <= strains <= 100%)	2,899
Soft core genes	(95% <= strains < 99%)	185
Shell genes	(15% <= strains < 95%)	35
Cloud genes	(0% <= strains < 15%)	31
Total genes	(0% <= strains <= 100%)	3,150

**Fig 1:**
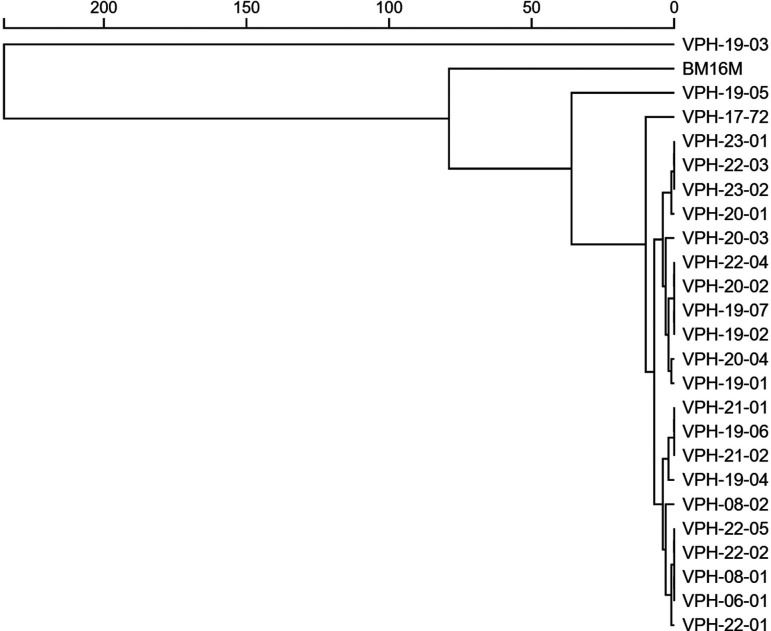
Dendrogram showing genetic relationships among *Brucella melitensis* isolates based on gene presence-absence. The scale at the top indicates genetic distance, with closer branches showing higher similarity between isolates.

**Fig 2:**
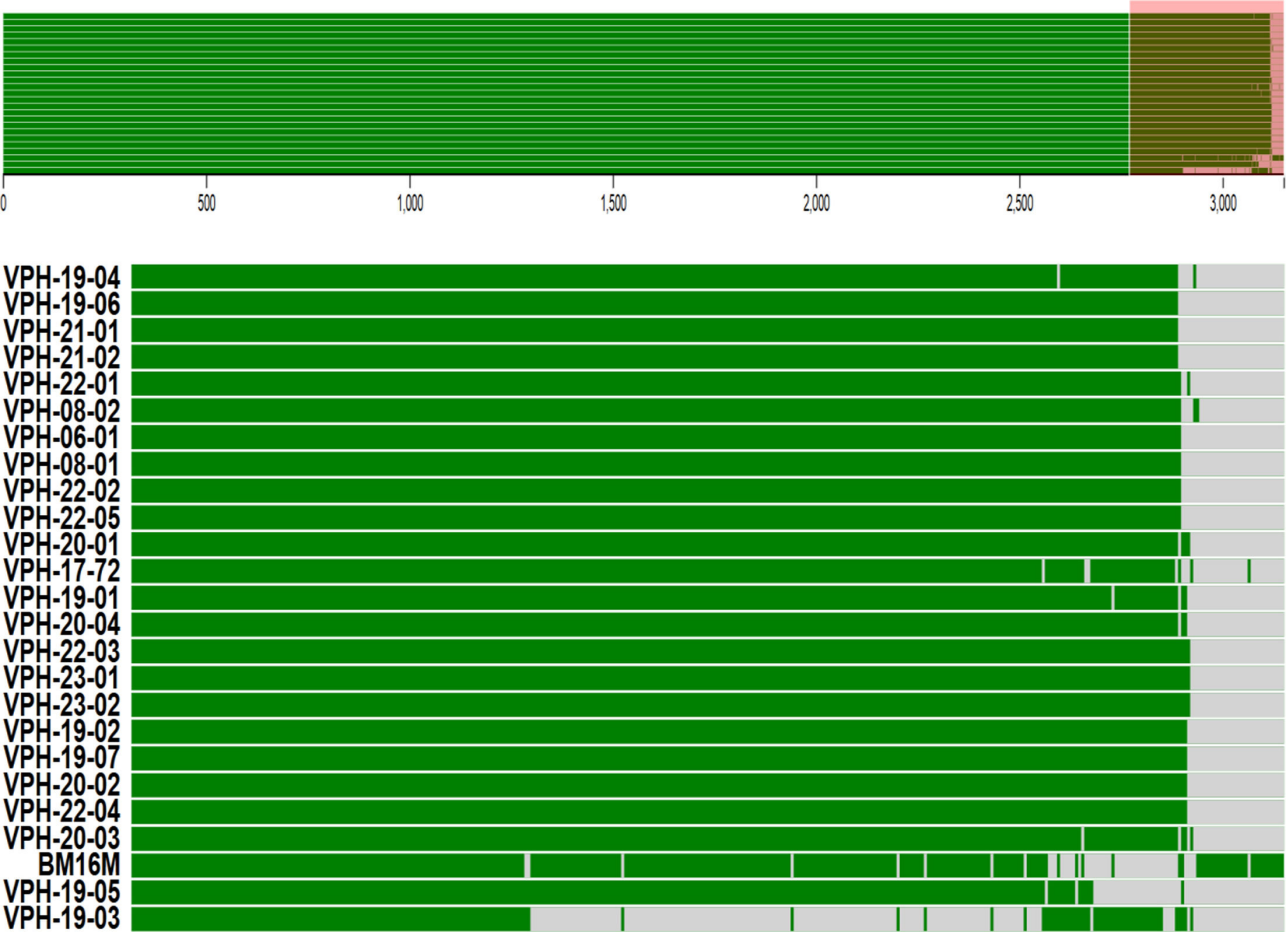
Comparative genome structure of *Brucella melitensis* strains based on gene presence-absence data. The top panel represents a two-dimensional whole-genome comparison, where green bars indicate the presence of genes and gray bars indicate their absence across different strains. The red-highlighted section at the right of the top panel shows a region of notable variability, which is expanded in the lower portion for a detailed view. Each row represents a different strain and the horizontal continuity of green bars denotes conserved regions, while interruptions reflect gene absences or strain-specific variations

The visualization of the pangenome composition further illustrated the conserved nature of the *B. melitensis* genome. The FriPan dendrogram (Fig. 1) identified five distinct clades among the Indian strains, with most isolates clustering into two main clades. The isolates VPH-19-03, VPH-19-05, and VPH-17-72 formed outgroups in the Fripan dendrogram due to the absence of certain genes, which distinguished them from the main clades within the dendrogram, as depicted with two-dimensional visualization of genomes in Fig. 2.

**Fig 3:**
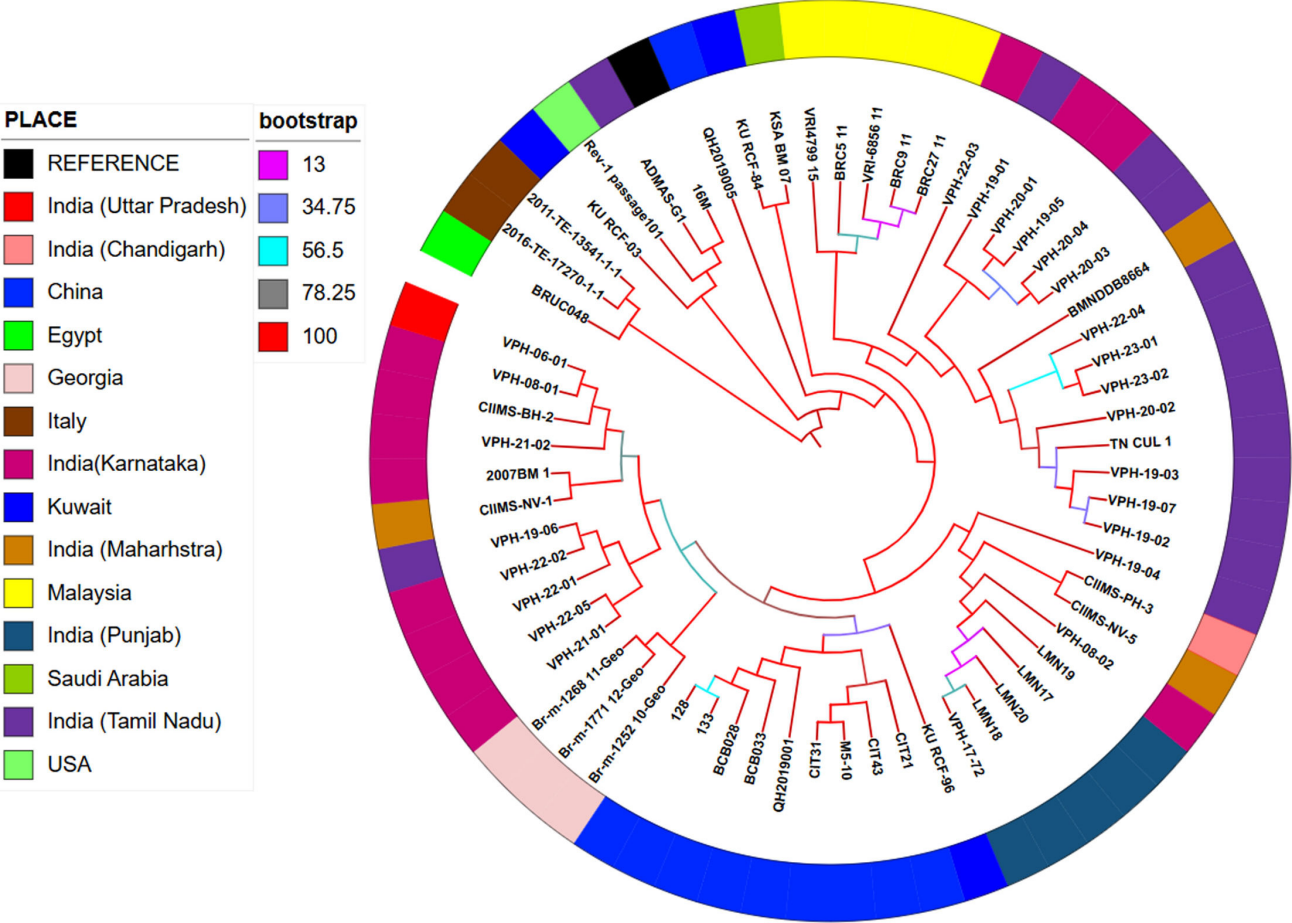
Core genome SNP-based maximum likelihood tree depicting the phylogenetic relationship of the study isolates to 38 strains from other countries (Branch color depicts bootstrap, Place of isolation is depicted by color strip).

The phylogenetic tree constructed with 38 strains from other countries revealed distinct clustering of Indian strains and those from other countries. Indian isolates did not cluster with isolates from China, Malaysia, Kuwait, or Saudi Arabia, which formed separate clades, indicating distinct phylogenetic lineages (Fig. 3).

### Minimum spanning tree analysis

**Fig 4:**
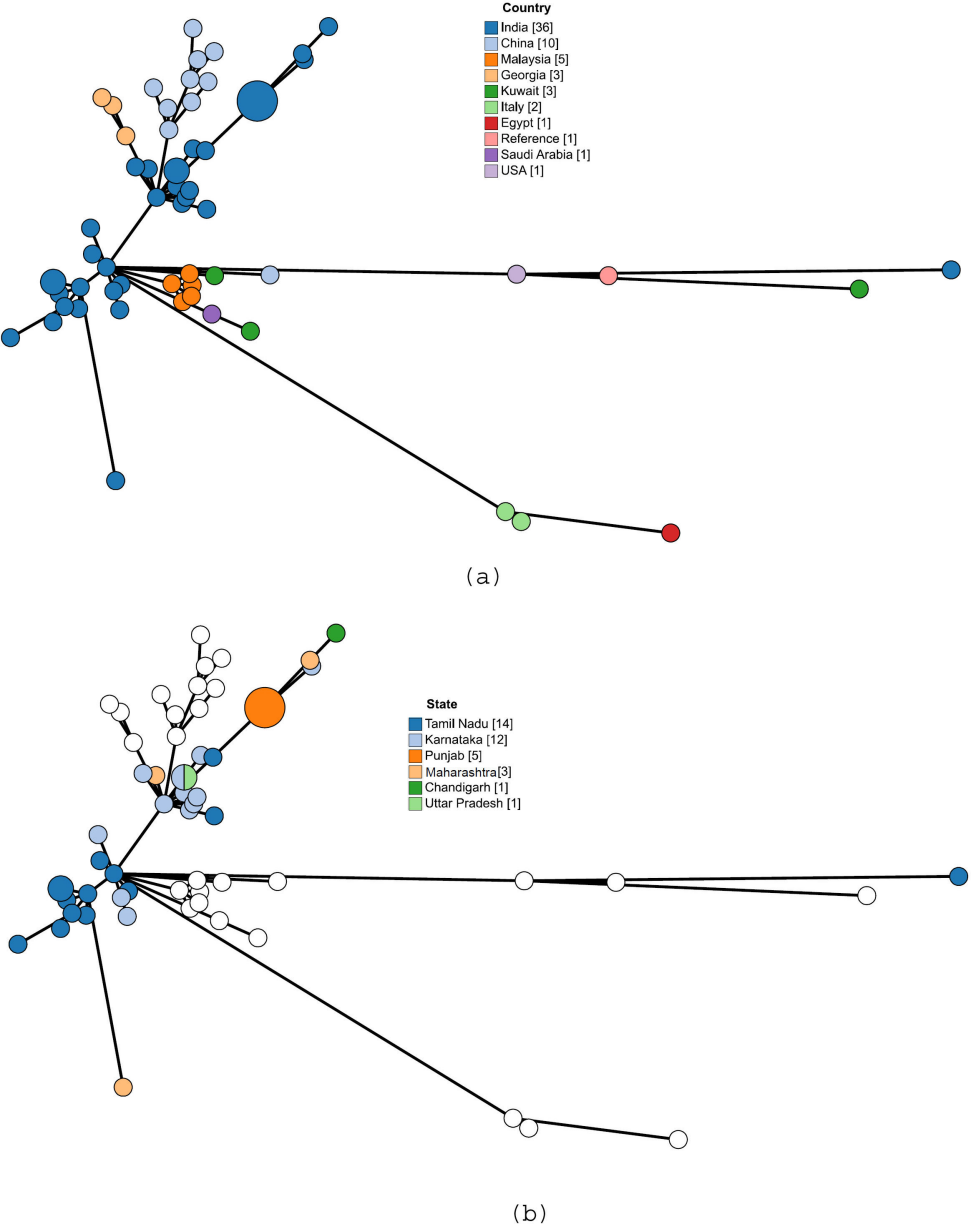
Minimum spanning tree depicting cgMLST profile of the *Brucella melitensis* isolates. Size of each circle proportionate to the number of isolates. (a) MST depicting the genetic relationships among 63 *B. melitensis* isolates, including 36 from India (blue) and 27 from other countries. Each node represents a unique isolate, with colors indicating geographic origin. (b) MST showing the genetic distribution of *B. melitensis* isolates in India, color-coded by state. White nodes represent global/reference strains.

The Minimum Spanning Tree (MST) analysis of 63 *Brucella melitensis* isolates, including 36 from India and 27 from global sources, revealed distinct genetic clustering patterns. Indian isolates (blue) formed a dense, interconnected cluster, indicating high genetic relatedness. Chinese isolates (red) and those from Malaysia, Georgia, Kuwait, and other regions formed separate branches, suggesting genetic divergence. A reference strain (black node) appeared as a distinct, unconnected entity (Fig. 4a). Some global isolates were positioned distantly, reflecting unique genetic variations. Within India (Fig. 4b), Tamil Nadu (blue) and Karnataka (light blue) isolates dominated the network with closely linked nodes, while Punjab (orange), Maharashtra (brown), and other states displayed separate clustering patterns. White nodes, representing isolates from global data sets or reference strains, diverged from Indian strains and appeared as distinct branches within the MST.

Among the Indian isolates, a total of 10 core genome sequence types (cgSTs) were identified, with cgST 670, 557, 565, and 573 being the most common, and cgST 670 being the most prevalent. Additional cgSTs included 225, 247, 556, 558, 1,185, and 1,167. Comparison with global strains revealed that Chinese isolates exhibited 10 distinct cgSTs among 10 strains, while Malaysia, Georgia, and Kuwait each had three cgSTs. The two isolates from Italy shared the same cgST, and the reference strain (cgST 218) (Table S1) showed no genetic overlap with the Indian isolates. The overall clustering pattern within the MST reflects cgST distribution, indicating high genetic relatedness among Indian strains and a clear separation from global isolates.

## DISCUSSION

Brucellosis is a significant zoonotic disease, necessitating early diagnosis and control measures. The pathogen is endemic in India, with a nationwide survey indicating seroprevalence rates of 11.55% in sheep and 5.37% in goats (41).

Virulence factor genes essential for the pathogenicity of *Brucella melitensis* are primarily involved in lipopolysaccharide (LPS) synthesis, intracellular survival, and secretion systems. Among the 43 identified virulence genes, the LPS operon (27 genes) plays a key role in immune evasion and intracellular persistence, allowing *B. melitensis* to avoid immune recognition (42). The type IV secretion system (*vir*B1-*vir*B12) secretes effector proteins that manipulate host cell processes, preventing phagosome-lysosome fusion and enabling bacterial replication within macrophages (43, 44). The bacterial TIR-domain-containing proteins, *btp*A and *btp*B, interfere with MyD88-dependent signaling, impairing pro-inflammatory cytokine responses and delaying host immune activation (45). The virulence gene profiles in our study were highly conserved across all Indian isolates, suggesting that *B. melitensis* relies on a stable set of pathogenicity determinants. Similar findings from other studies reinforce the significance of these genes in pathogenesis and host adaptation (11, 46). This conservation aligns with global reports indicating a highly stable pathogenicity profile across different geographical regions (14, 15, 47).

Few studies have conducted genome analysis and comparative genomics of Indian *B. melitensis* strains. Pan-genome analysis revealed a highly conserved core genome with 2,899 core genes and only 185 accessory genes among the 24 isolates analyzed. This conservation aligns with previous findings of low genetic variability within *B. melitensis* (48). However, the identification of unique gene profiles in isolates such as VPH-19-03 suggests that regional adaptations and niche-specific evolutionary pressures may contribute to genetic differentiation. Such findings emphasize the dynamic nature of bacterial genomes in response to environmental and host-specific factors (49, 50). Two-dimensional scaling indicated five clades among the Indian strains, and clustering of strains from the same regions were observed. This highlights the genetic stability within a particular region and the possibility for differentiating strains based on whole-genome sequence data. Multilocus sequence typing (MLST) showed that all 24 Indian strains belonged to ST8. Identification of five different cgMLST types aligns with the clustering analysis. Phylogenetic analyses further supported the distinct clustering of Indian strains, separate from those of other countries. Phylogenetic comparisons with 38 global strains showed that Indian isolates did not cluster with strains from China, the Middle East, or Southeast Asia, supporting restricted genetic exchange and localized evolution in *B. melitensis*. This finding is crucial for brucellosis epidemiology, as it indicates that outbreaks in India are likely driven by endemic transmission rather than frequent introductions from other regions. Similar studies using SNP-based whole-genome phylogenetics have also reported distinct phylogenetic separation of *B. melitensis* strains by geographic origin, highlighting the role of regional selection pressures in shaping bacterial evolution (11, 51). Phylogenetic analysis of Indian strains in our study demonstrated geographical clustering and showed strong correlation with cgMLST, consistent with findings by (11, 46). The minimum spanning tree (MST) analysis of *B. melitensis* isolates closely mirrored phylogenetic relationships, effectively capturing genetic relatedness and geographic distribution patterns. The clustering of strains within the MST indicated high genetic relatedness among Indian isolates and a clear separation from global strains. This observation aligns with previous studies that have demonstrated regional clustering of *B. melitensis* strains (52–54). These insights into the genomic epidemiology of *B. melitensis* are essential for the development of more effective diagnostic tools, which can improve early detection and control of brucellosis outbreaks. Moreover, the identification of conserved virulence genes and stable sequence types provides promising targets for vaccine development, which could lead to more effective prevention measures. Additionally, the genetic information uncovered in this study has the potential to inform therapeutic interventions, guiding the development of treatments that are tailored to the specific genetic makeup of regional *B. melitensis* strains.

The absence of specific genes in *B. melitensis* is a significant factor in the bacterium’s genome reduction, a process involving the loss of non-essential genes over time. Genome reduction is a common evolutionary strategy among intracellular pathogens and symbionts, leading to streamlined genomes highly specialized for their specific niches (55). This phenomenon results in the loss of genes involved in diverse metabolic pathways, stress responses, and regulatory networks. For instance, genes encoding for enzymes, such as glycine cleavage system H protein, 5-deoxy-glucuronate isomerase, and uronate isomerase, which are involved in amino acid degradation and carbohydrate metabolism, may be deemed non-essential if the host provides sufficient nutrients. The absence of these genes in *B. melitensis* suggests a reliance on the host for specific nutrients, reducing the metabolic burden on the bacterium’s genome and contributing to genome streamlining (55, 56).

The loss of genes involved in stress responses, such as the sulfur carrier protein *fdh*D and ABC transporter systems (e.g., y*cj*O, o*us*W), indicates an adaptation to the relatively stable intracellular environment of the host. These genes are critical for surviving external stress conditions, but within the host, *B. melitensis* experiences less environmental variability and fewer oxidative stresses. Thus, these genes become redundant, and their loss aids in genome reduction by eliminating unnecessary genetic material (57). Although essential for ribosome function and protein synthesis, certain ribosomal RNA modification genes, such as the ribosomal RNA large subunit methyltransferase E, may be lost if redundant or if alternative pathways suffice. Genome reduction in this context involves retaining only the most efficient and necessary components for protein synthesis, reflecting a highly specialized adaptation to the host environment (56).

Several genes regulating virulence factors, such as the DNA replication inhibitor toxin *soc*B and various regulatory proteins (e.g., hydrogen peroxide-inducible genes activator, leucine-responsive regulatory protein), may be lost if their functions are compensated by other mechanisms or if they are not essential for survival within the host. The absence of these genes can lead to a streamlined genome that focuses on essential virulence factors, enhancing the bacterium’s efficiency in host colonization and infection (58). ABC transporter proteins (e.g., *ycj*O, *opp*D, *opp*C) and other membrane-associated proteins are often lost during genome reduction if the host environment sufficiently supports the bacterium’s nutrient and ion needs. The absence of these transport systems reduces the genomic and metabolic burden, allowing *B. melitensis* to maintain a minimalistic yet effective set of transport mechanisms necessary for survival within the host (55).

Genome reduction in *B. melitensis* leads to the loss of non-essential genes involved in metabolic versatility, stress response, and regulatory networks, resulting in a more specialized pathogen with a narrow ecological niche. This reduction might limit the bacterium’s ability to adapt to diverse environments and host conditions, impacting its metabolic flexibility and stress resilience (42, 59). However, streamlining the genome can enhance the efficiency of the remaining pathways, providing a selective advantage within specific niches, such as host cells, where the pathogen may exhibit increased survival. Additionally, the absence of horizontal DNA transfer mechanisms and mobile genetic elements further stabilizes the genome, contributing to host adaptation and virulence through pseudogenization and single-nucleotide polymorphisms (60). This evolutionary trade-off reflects a balance between adaptability and specialization, influencing the overall pathogenicity and survivability of *B. melitensis*.

### Conclusion

The integration of virulence gene analysis, multilocus sequence typing (MLST), pangenome, and phylogenetic approaches provides a robust and multifaceted understanding of the genetic diversity and evolutionary dynamics of *Brucella melitensis*. By employing these advanced genomic techniques, the study reveals the genetic stability of sequence type ST8 among Indian strains, which appears to be conserved despite varying environmental and host-related pressures. This stability is particularly significant in the context of ability of *B. melitensis* to cause brucellosis, a disease of substantial public health and economic concern in India. The identification of 43 key virulence genes further underscores the critical role these genetic elements play in the pathogen’s ability to infect and cause disease.

The research findings suggest that regional adaptations, driven by unique environmental conditions and host interactions, as well as niche-specific evolutionary pressures, are contributing factors to the genetic differentiation observed among Indian isolates. These adaptations likely reflect a complex interplay between the bacterium and its environment, which may influence its virulence and transmission dynamics. Understanding these factors is crucial not only for grasping the local epidemiology of brucellosis but also for informing global strategies to combat this zoonotic disease.

Beyond its implications for India, this research contributes to the global understanding of *B. melitensis* diversity and evolution, offering valuable insights that can be applied to other regions where brucellosis remains a significant threat. By enhancing the broader scientific community’s knowledge of the genetic and evolutionary underpinnings of this pathogen, the study lays the groundwork for more coordinated and informed efforts to manage and ultimately eradicate brucellosis worldwide.

## Data Availability

The Whole Genome Sequences of the study isolates have been deposited in GenBank with relevant metadata under accession nos. JAYWOX000000000, JAYWOY000000000, JAYWOZ000000000, JAYWPA000000000, JAYWPB000000000, JAYWPC000000000, JAYWPD000000000, JAYWPF000000000, JAYWPG000000000, JAYWPH000000000, JAYWPI000000000, JAYWPJ000000000, JAYWPK000000000, JAYWPL000000000, JAYWPM000000000, JAYWPN000000000, JAYWPK000000000, JAYWPO000000000, JAYWPP000000000, JAYWPQ000000000, JAYWPR000000000, JAYWPS000000000 and JAYWPT000000000.

## SUPPLEMENTAL MATERIAL

Table S1 spectrum.02647-24-s0001.docx Core genome sequence types and geographic distribution of *Brucella melitensis* isolates.

ASM does not own the copyrights to Supplemental Material that may be linked to, or accessed through, an article. The authors have granted ASM a non-exclusive, world-wide license to publish the Supplemental Material files. Please contact the corresponding author directly for reuse.
