# Supplementary material for: Genomic insights into Brucella melitensis in India: stability of ST8 and the role of virulence genes in regional adaptations
Source: Microbiol Spectr. 2025 Apr 24;13(6):e02647-24. doi: 10.1128/spectrum.02647-24 (PMC12131837; doi:10.1128/spectrum.02647-24)
Supplement: Table S1 — Core genome sequence types and geographic distribution of Brucella melitensis isolates. [file spectrum.02647-24-s0001.docx]

**Table S1:** Core Genome Sequence Types (cgST) and Geographic Distribution of *Brucella melitensis* Isolates

| **Group** | **Strain** | **Place** | **cgST** |
| --- | --- | --- | --- |
| Group0 | BMNDDB8664 | India (Maharashtra) | 1185 |
| Group0 | TN_CUL_1 | India (Tamil Nadu) | 670 |
| Group0 | VPH-19-01 | India (Tamil Nadu) | 670 |
| Group0 | VPH-19-02 | India (Tamil Nadu) | 670 |
| Group0 | VPH-19-03 | India (Tamil Nadu) | 670 |
| Group0 | VPH-19-05 | India (Karnataka) | 670 |
| Group0 | VPH-19-07 | India (Tamil Nadu) | 670 |
| Group0 | VPH-20-01 | India (Karnataka) | 670 |
| Group0 | VPH-20-02 | India (Tamil Nadu) | 670 |
| Group0 | VPH-20-03 | India (Tamil Nadu) | 670 |
| Group0 | VPH-20-04 | India (Tamil Nadu) | 670 |
| Group0 | VPH-22-03 | India (Karnataka) | 670 |
| Group0 | VPH-22-04 | India (Tamil Nadu) | 670 |
| Group0 | VPH-23-01 | India (Tamil Nadu) | 670 |
| Group0 | VPH-23-02 | India (Tamil Nadu) | 670 |
| Group1 | 2007BM_1 | India (Karnataka) | 1167 |
| Group1 | CIIMS-BH-2 | India (Karnataka) | 557 |
| Group1 | CIIMS-NV-1 | India (Maharashtra) | 565 |
| Group1 | VPH-06-01 | India (Uttar Pradesh) | 557 |
| Group1 | VPH-08-01 | India (Karnataka) | 557 |
| Group1 | VPH-19-06 | India (Tamil Nadu) | 557 |
| Group1 | VPH-21-01 | India (Karnataka) | 557 |
| Group1 | VPH-21-02 | India (Karnataka) | 557 |
| Group1 | VPH-22-01 | India (Karnataka) | 557 |
| Group1 | VPH-22-02 | India (Karnataka) | 557 |
| Group1 | VPH-22-05 | India (Karnataka) | 565 |
| Group10 | KSA_BM_07 | Saudi Arabia | 567 |
| Group10 | KU_RCF-84 | Kuwait | 486 |
| Group11 | BRUC048 | Egypt | 484 |
| Group12 | CIT21 | China | 568 |
| Group13 | KU_RCF-03 | Kuwait | 489 |
| Group14 | KU_RCF-96 | Kuwait | 1147 |
| Group15 | QH2019005 | China | 673 |
| Group16 | Rev-1_passage101 | USA | 560 |
| Group17 | VPH-19-04 | India (Tamil Nadu) | 225 |
| Group2 | LMN17 | India (Punjab) | 573 |
| Group2 | LMN18 | India (Punjab) | 573 |
| Group2 | LMN19 | India (Punjab) | 573 |
| Group2 | LMN20 | India (Punjab) | 573 |
| Group2 | VPH-08-02 | India (Karnataka) | 225 |
| Group2 | VPH-17-72 | India (Punjab) | 573 |
| Group3 | 128 | China | 277 |
| Group3 | 133 | China | 1136 |
| Group3 | BCB028 | China | 1135 |
| Group3 | BCB033 | China | 1134 |
| Group3 | QH2019001 | China | 672 |
| Group4 | BRC27_11 | Malaysia | 546 |
| Group4 | BRC5_11 | Malaysia | 546 |
| Group4 | BRC9_11 | Malaysia | 546 |
| Group4 | VRI4799_15 | Malaysia | 547 |
| Group4 | VRI-6856_11 | Malaysia | 545 |
| Group5 | Br-m-1252_10-Geo | Georgia | 1151 |
| Group5 | Br-m-1268_11-Geo | Georgia | 1152 |
| Group5 | Br-m-1771_12-Geo | Georgia | 1150 |
| Group6 | CIT31 | China | 569 |
| Group6 | CIT43 | China | 570 |
| Group6 | M5-10 | China | 272 |
| Group7 | 16M | Reference | 218 |
| Group7 | ADMAS-G1 | India (Tamil Nadu) | 247 |
| Group8 | 2011-TE-13541-1-1 | Italy | 652 |
| Group8 | 2016-TE-17270-1-1 | Italy | 652 |
| Group9 | CIIMS-NV-5 | India (Maharashtra) | 556 |
| Group9 | CIIMS-PH-3 | India (Chandigarh) | 558 |
